# Impact of Heat Stress on the Predatory Ladybugs *Hippodamia variegata* and *Propylaea quatuordecimpunctata*

**DOI:** 10.3390/insects13030306

**Published:** 2022-03-20

**Authors:** Qing Yang, Jinping Liu, Kris A. G. Wyckhuys, Yizhong Yang, Yanhui Lu

**Affiliations:** 1 College of Horticulture and Plant Protection, Yangzhou University, Yangzhou 225007, China; yangqingzzr@126.com; 2 State Key Laboratory for Biology of Plant Diseases and Insect Pests, Institute of Plant Protection, Chinese Academy of Agricultural Sciences, Beijing 100193, China; 13720142405@163.com (J.L.); k.wyckhuys@uq.edu.au (K.A.G.W.)

**Keywords:** biological control, IPM, temperature stress, sustainable agriculture, climate adaptability, antioxidant response

## Abstract

**Simple Summary:**

As poikilotherms, insects are sensitive to ambient environmental conditions; therefore, it is important to gauge how heat stress affects their survival and fitness. The ladybeetles *Hippodamia variegata* (Goeze) and *Propylaea quatuordecimpunctata* (Linnaeus) are key natural enemies within cotton fields in Xinjiang Province, China. This study investigated the effects of different temperatures (i.e., 32, 35, and 38 °C) on the survival, reproduction, predation, and antioxidant capacity of adult ladybugs. Laboratory assays showed that elevated temperatures (i.e., 35 and 38 °C) impacted *P. quatuordecimpunctata* survival and reproduction to a greater extent than that of *H. variegata*. At all experimental temperatures, *H. variegata*’s predation rate on aphid prey surpassed that of *P. quatuordecimpunctata*. Yet, prey consumption rates of *H. variegata* were highest at 35 °C, while those of *P. quatuordecimpunctata* gradually decreased with higher temperatures. Lastly, superoxide dismutase (SOD), catalase (CAT), peroxidases (POD), glutathione-s-transferases (GSTs), total antioxidant capacity (T-AOC), and protein content in both ladybugs were significantly affected by ambient temperature. By assessing the thermal biology of individual ladybug species, laboratory assays can thus explain their spatiotemporal distribution and inform strategies to enhance biological control under conditions of global warming or extreme weather events.

**Abstract:**

In cotton-growing regions of northwestern China, *Hippodamia variegata* (Goeze) and *Propylaea quatuordecimpunctata* (Linnaeus) (Coleoptera: Coccinellidae) are key natural enemies of hemipteran pests. As only *H. variegata* can be encountered in hot, arid production areas, the thermal responses and climatic adaptability of both species likely differ substantially. In this study, we assessed the survival, longevity, fecundity, prey consumption rate, and antioxidant capacity of both species under laboratory conditions at 32–38 °C. The (negative) impacts of elevated temperatures (i.e., 35 and 38 °C) on adult survival and reproduction were more pronounced for *P. quatuordecimpunctata* than for *H. variegata*. Similarly, high temperatures exhibited the strongest negative impacts on the prey consumption rates of *P. quatuordecimpunctata*. At elevated temperatures, superoxide dismutase and catalase activity increased, while glutathione-S-transferases activity decreased for both species. However, for *P. quatuordecimpunctata*, peroxidase activity and total antioxidant capacity progressively declined. Antioxidant responses thus constitute a key physiological adaptation of ladybugs to heat stress, reflecting a superior thermal tolerance of *H. variegata*. Our work emphasizes how laboratory assays can explain spatiotemporal distribution patterns of individual ladybugs and inform strategies to bolster their ensuing biological control under conditions of global warming or extreme weather events.

## 1. Introduction

As poikilotherms, insects are sensitive to ambient environmental conditions. Temperature hereby affects growth and development, physiology, behavior, and geographical distribution of beneficial insects and agricultural pests alike [1,2,3,4]. Given that countless insect species contribute to natural biological control [5,6], it is crucial to investigate temperature-mediated impacts on their development and ensuing population dynamics. Ladybugs are globally important natural enemies that contribute to the regulation of multiple crop pests such as mites or aphids [7,8]. Laboratory studies have shown how elevated temperatures affect growth, development, and survival of both immature and adult stages of various ladybug species [9,10,11]. Impacts are species-specific, depend upon the range of experimental temperatures [11], and equally involve other life history parameters such as fecundity [12,13]. Another key (temperature-dependent) variable is ladybugs’ contribution to biological control [14]. Laboratory assays have demonstrated how temperature modulates predation rates of various ladybug species and development stages as well as targets prey items, e.g., *Exochomus nigripennis* (Erichson) larvae on *Gossyparia spuria* (Modeer) [11], *Cheilomenes sexmaculata* (Fabricius) larvae and adults on *Megoura japonica* (Matsumura) [15], or *Micraspis discolor* (Fabricius) larvae on *Brevicoryne brassicae* (L.) [16]. Hence, to sustain or promote ladybug-mediated biological control in (temporally) hot production settings or under climate change scenarios, it is essential to gain a better understanding of these temperature-related impacts [17,18].

Elevated temperatures also result in physiological changes, e.g., involving oxidative damage through reactive oxygen species (ROS) [19,20]. To mitigate ROS-inflicted damage, insects deploy antioxidant defenses against different reactive chemicals using catalase (CAT), peroxidase (POD), super-oxide dismutase (SOD) and glutathione-S-transferases (GSTs) [21,22]. These defenses lead to an overall organismal ability to resist stress, which is captured by total antioxidant capacity (T-AOC) [23]. In ladybugs, such as *Harmonia axyridis* (Pallas) or *Propylaea japonica* (Thunberg), T-AOC and GST activity decreases with surging temperatures, while SOD activity exhibits more variable effects depending upon the experimental temperature regime [24,25]. Lastly, through the above physiological and behavioral changes, temperature mediates the seasonal abundance, dispersal patterns, and geographical distribution of herbivorous insects [26] and ladybug predators alike [27,28,29]. This can lead to a mismatch between predator and prey population phenology and potentially can trigger pest outbreaks.

In northwestern China, cotton is cultivated in a broad suite of climatically distinct regions. Cotton production areas are situated in hot, arid settings (up to 40 °C, according to the China Meteorological Administration, 1981~2010) but also in colder environments, e.g., in the northern range of the Tianshan Mountains [30,31,32]. Equally, the cotton growing season is typified by important geographically variable temperature increases. In the local cotton crop, the two main predatory ladybugs, *Hippodamia variegata* (Goeze) and *Propylaea quatuordecimpunctata* (Linnaeus)*,* contribute to the biological control of economical pests, i.e., aphids, mites, and thrips [33,34,35]. Yet, while *H. variegata* is widely distributed, *P. quatuordecimpunctata* is only found in northern-most (colder) areas. Whether this difference in geographical distribution is related to differences in the species’ thermal tolerance remains to be determined.

In this study, we used laboratory assays to assess the effects of elevated temperatures (i.e., 32–38 °C) on the survival, reproduction, predation ability, and antioxidant capacity of *H. variegata* and *P. quatuordecimpunctata*. Our findings not only yielded baseline information on the temperature-dependent development of ladybugs but also provide a theoretical basis for the development of biological control strategies and ladybug conservation tactics.

## 2. Materials and Methods

### 2.1. Insects Sources

Individuals of *H. variegata* and *P. quatuordecimpunctata* were collected from cotton plots (pesticide-free) of the experimental field station at Shihezi University (44.32° N, 85.92° E) (Shihezi, Xinjiang Uygur Autonomous Region, China) on 27 July 2019. Diagnostic keys were used to confirm species’ identity [36]. Next, field-caught individuals were transferred to the Langfang experimental station, Chinese Academy of Agricultural Sciences (CAAS; 39.53° N, 116.70° E) in Langfang, Hebei Province. Ladybugs were reared in a plastic container (diameter: 8 cm; height: 11.5 cm) within a controlled climate chamber (RXZ-500D, Ningbo Jiangnan Instrument Factory, Ningbo, China) at 32 ± 1 °C, 70 ± 5% RH, and 16:8 h (L:D) photoperiod. On a daily basis, ladybugs were fed ad libitum with cotton aphids (*Aphis gossypii* Glover) and the F_1_ progeny was used for experimentation. Cotton aphids were also collected from cotton fields of the experimental field station at Shihezi University, transferred to the laboratory, and subsequently reared on *Cucurbita pepo* L. (Xinzaoqing seed, Tianjin City Ji Nong Seed Co., Ltd., Tianjin, China). Aphids were maintained within (55 × 35 × 50 cm) screened cages in a greenhouse at 28–30 °C, 50 ± 5% RH, and 16:8 h (L:D) photoperiod.

### 2.2. Experimental Temperature Range

All subsequent experimental assays were performed in the lab and carried out in climatic chambers with temperatures of 32 (as control), 35 (medium–high temperature), and 38 °C (high temperature), which mirror the locally prevailing temperatures during the cotton growing season in northwestern China.

### 2.3. Adult Survival and Reproduction

F_1_ adults of *H. variegata* and *P. quatuordecimpunctata* < 12 h of age were removed from the rearing colony, placed within mesh-covered plastic recipients (diameter: 8 cm; height: 11.5 cm), and fed cotton aphids ad libitum. Recipients were kept at 32 °C and adults were allowed to freely oviposit. Every day, adults were transferred into a new plastic container, and deposited eggs were left in place until hatching. Newly emerged larvae were then transferred into new containers and fed ad libitum with cotton aphids until pupation. Upon pupal emergence, 30 pairs of F_2_ adults (<12 h of age) were randomly selected and placed in climatic chambers at 32, 35, or 38 °C, 70 ± 5% RH, and 16:8h (L:D) photoperiod, respectively. All of the climatic chambers before the experiment used HOBO instruments to ensure a constant temperament. Each pair of ladybugs was placed in Petri dishes (diameter: 15 cm; height: 2 cm), containing a water-moistened cotton pad and a *Cucurbita pepo* leaf with approximately 1000 aphids. On a daily basis, ladybug survival and the number of deposited eggs were recorded, and the adults were moved into a new plastic Petri dish. Observations continued until all adults died. There were three replicates for each temperature condition (i.e., treatment) and ten pairs of ladybugs per replicate.

### 2.4. Adult Predation

According to Jermy et al. [37], we assessed the predation rate of *H. variegata* and *P. quatuordecimpunctata* at the three experimental temperatures and above climatic conditions (70 ± 5% RH, 14:10 (L:D) photoperiod). At the onset of the experiment, adult females of either ladybug species (<12 h of age) were individually starved for 24 h. Next, each individual ladybug was transferred onto an excised *Cucurbita pepo* leaf disc (equal to the area of the Petri dish), placed on a layer of 1% agarose (to slow water loss) within a Petri dish (diameter: 15 cm; height: 2 cm). Experimental areas contained different numbers of *A. gossypii* prey, i.e., 50, 100, 150, 200, 250, 300, or 350 individuals of 4th instar aphids. At each experimental temperature, a total of five ladybug adults (i.e., replicates) were individually exposed to a given number of prey items for 24 h. Next, we recorded the number of aphids consumed by each ladybug adult.

### 2.5. Antioxidant Responses

A pair of adults (<12 h of age) of either ladybug species was placed within a Petri dish (diameter: 15 cm; height: 2 cm) and subjected to 24 h on the experimental temperatures, i.e., 32, 35, and 38 °C. Next, live, healthy adults were chosen, quickly immersed in liquid nitrogen, and then they were stored in an −80 °C refrigerator until further laboratory testing. Next, frozen individuals were placed within extraction buffer solution (EBS) at a ratio of 1 ml EBS for each 0.1 g of body tissue. Samples were placed in a cold mortar, homogenized with liquid nitrogen, and crude extracts were centrifuged at 4 °C and 10,000× *g* for 10 min. The supernatant was then centrifuged under the above conditions to determine the antioxidant capacity. Three replicates were used for each ladybug species at each temperature treatment.

The activity levels of the different antioxidant enzymes (i.e., SOD, CAT, POD, and GSTs) were determined using commercial assay kits (Beijing Solarbio Science & Technology Co., Ltd., Beijing, China) following the manufacturer’s instructions. Absorbance was recorded using a UV–Visible Spectrophotometer (UNICO Instrument Co., Ltd., Shanghai, China), with the activity of SOD detected at 560, CAT at 240, POD at 470, and GSTs at 340 nm. The T-AOC activities were determined using commercial assay kits (Beijing Solarbio Science & Technology Co., Ltd., Beijing, China) following the manufacturer’s instructions, with recordings made at 593 nm. Lastly, protein concentration was measured using the Easy II Protein Quantitative Kit (BCA) (TransGen Biotech, Beijing, China) with readings made at 562 nm.

### 2.6. Data Analysis

Functional responses were described using a two-stage analysis [38]. Based upon the relationship between initial prey density and the actual number of consumed prey, the type of functional response curve was first determined following the formula below:

*N*_a_/*N*_0_ = exp(*P*_0_ + *P*_1_*N*_0_ + *P*_2_*N*_0_^2^ + *P*_3_*N*_0_^3^)/1 + exp(*P*_0_ + *P*_1_*N*_0_ + *P*_2_*N*_0_^2^ + *P*_3_*N*_0_^3^)

where *N*_a_ is the number of consumed prey items, *N*_0_ is the initial prey number, and *P*_0_ (intercept), *P*_1_ (linear), *P*_2_ (quadratic), and *P*_3_ (cubic) are coefficients estimated through the maximum likelihood method. Next, the shape of the functional response curve (e.g., type II or III) was determined as per De Clercq et al. [39]. While the prey consumption rate exhibits a curvilinear increase for type II functional responses, a sigmoid curve is recorded for type III responses. The related equations are described by:

*N*_a_ = *aNT*/(1 + *aNT*_h_) (type II)

*N*_a_ = (*d* + *bN*)*NT*/[1 + *cN* + (*d* + *bN*)*NT*_h_] (type III)

where *N*_a_ is the number of prey, *N* is prey density, *a* is attack rate, *T*_h_ is prey handling time, *T* is the time available for the predator to find the prey (1 d), and *b*, *c*, and *d* are constants [38].

One-way analysis of variance (ANOVA) was used to analyze the effect of temperature on adult longevity, fecundity, and antioxidant titers of ladybugs. Tukey’s test was used to determine differences between different temperatures for the same ladybug (*p* < 0.05), and the differences between different ladybugs at the same temperature were analyzed by the Student’s *t*-test (*p* < 0.05). Survival curves of both species were analyzed by the Kaplan–Meier log-rank test. All statistical analyses were conducted using SPSS 25.0 software and the R programming language version 2.0.1, while charts were generated using SigmaPlot 12.5 and OriginPro 9.0.

## 3. Results

### 3.1. Adult Survival and Reproduction

**Figure 1:**
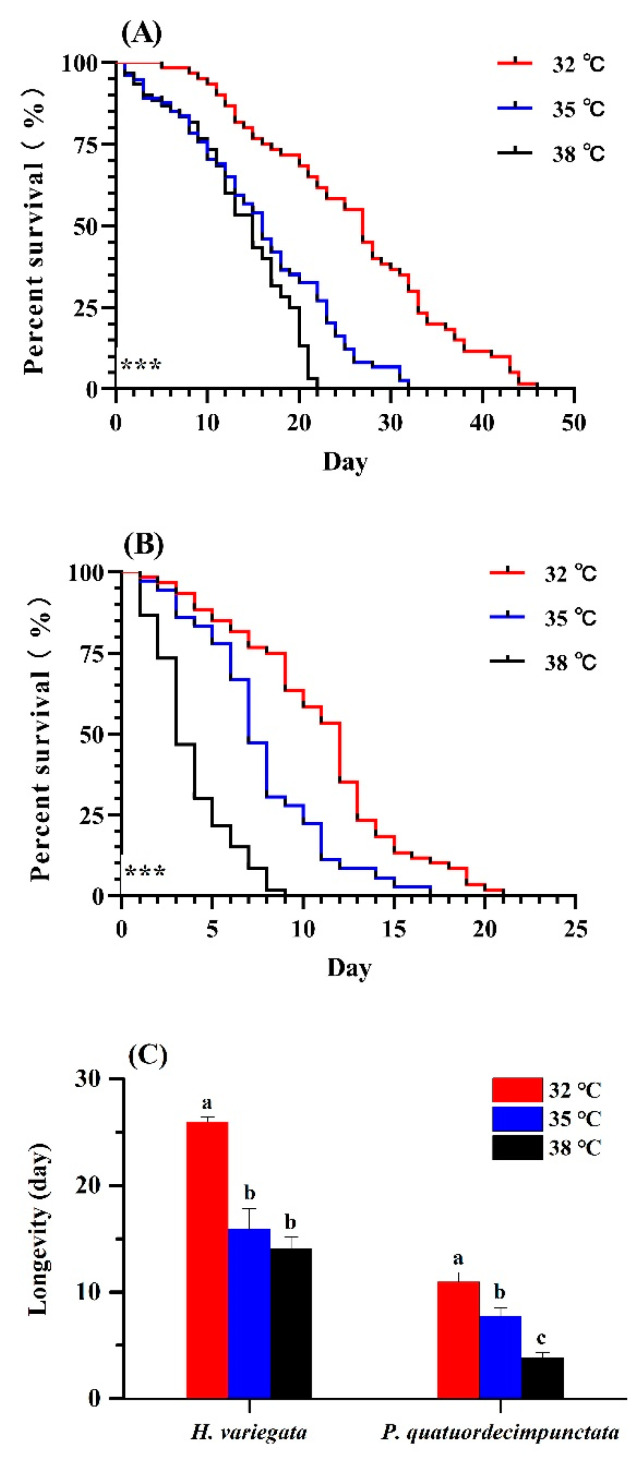
Survival curves of adult *Hippodamia variegata* (**A**) and *Propylaea quatuordecimpunctata* (**B**) at different temperatures. (**C**) Adult longevity of both species. Survival statistics were calculated using the Kaplan–Meier survival curve and compared using the log-rank test (individuals = 60, *** *p* < 0.001). For each species, different letters above the bars indicate statistically significant differences (ANOVA; Tukey’s post hoc test; *p* < 0.05).

Survival of *H. variegata* and *P. quatuordecimpunctata* adults was affected by temperature (log-rank test: *χ*^2^ = 64.82, *df* = 2, *p* < 0.001; *χ*^2^ = 102.69, *df* = 2, *p* < 0.001, respectively; Figure 1A,B). At all temperatures, *H. variegata* attained the highest survival (log-rank test: 32 °C: *χ*^2^ = 77.77, *df* = 1, *p* < 0.001; 35 °C: *χ*^2^ = 43.12, *df* = 1, *p* < 0.001; 38 °C: *χ*^2^ = 36.36, *df* = 1, *p* < 0.001). At 38 °C, *P. quatuordecimpunctata* survival declined significantly on the 3rd day compared to other temperatures (Tukey’s test: *F*_2, 6_ = 252.33, *p* < 0.001) (Figure 1B). Hence, elevated temperatures negatively impacted *P. quatuordecimpunctata* more than that of *H. variegata*.

The longevity of *H. variegata* and *P. quatuordecimpunctata* were equally affected by temperature (Tukey’s test: *F*_2, 6_ = 38.28, *p* < 0.001; *F*_2, 6_ = 29.51, *p* = 0.001, respectively). For *H. variegata*, only an initial rise in temperature to 35 °C, lowered longevity (*t*-test: *t* = 8.76, *df* = 1, *p* = 0.001) (Figure 1C), while further temperature increases also lowered the longevity of *P. quatuordecimpunctata*, declining from 10.96 (32 °C) to 7.72 d (35 °C) to 3.83 d at 38 °C, respectively (*t*-test: 32–35 °C: *t* = 3.40, *df* = 1, *p* = 0.027; 35–38 °C: *t* = 7.06, *df* = 1, *p* = 0.002) (Figure 1C).

**Figure 2:**
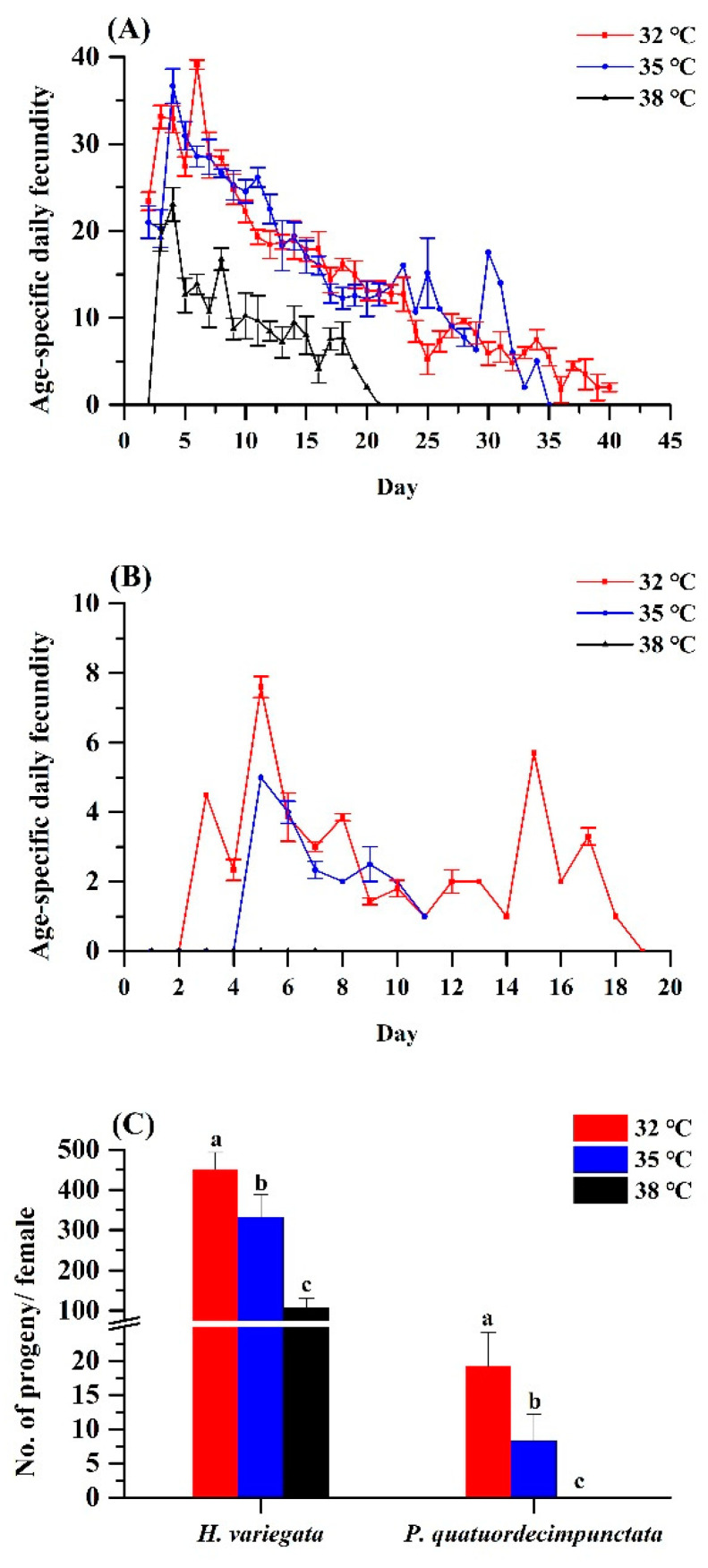
Age-specific fecundity of adult *Hippodamia variegata* (**A**) and *Propylaea quatuordecimpunctata* (**B**) at different temperatures. (**C**) Fecundity of both species. For each species, different letters above the bars indicate statistically significant differences among temperatures (ANOVA; Tukey’s post hoc test; *p* < 0.05).

Elevated temperature equally affected age-specific and total fecundity of both ladybug species (Figure 2A,B). For *H. variegata*, oviposition rates dropped from 450.77 to 332.19 to 107.38 eggs per female as temperatures rose from 32 over 35 to 38 °C (Tukey’s test: *F*_2, 6_ = 50.21, *p* < 0.001). Peak oviposition shifted from day 6 at 32 °C and on day 4 at 35 and 38 °C. For *P. quatuordecimpunctata*, total oviposition was significantly lower than *H. variegata* at all temperatures. Oviposition rates of *P. quatuordecimpunctata* declined from 19.19 (32 °C) to 8.27 (35 °C) to 0 at 38 °C, respectively (Tukey’s test: *F*_2, 6_ = 20.70, *p* = 0.002). For all temperatures, peak oviposition was recorded on day 5 (Figure 2).

### 3.2. Functional Response

**Figure 3:**
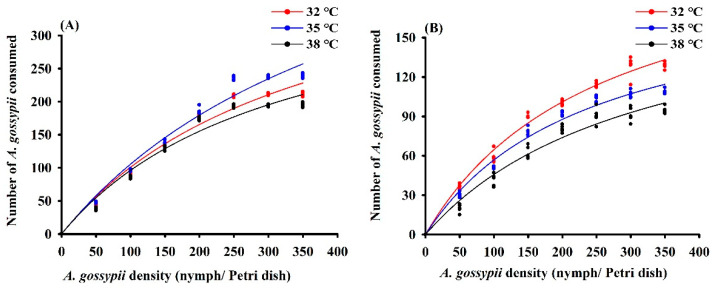
Functional response curves of adult female *Hippodamia variegata* (**A**) and *Propylaea quatuordecimpunctata* (**B**) to 4th instar *Aphis gossypii* at different temperatures.

Across temperature regimes, *H. variegata* and *P. quatuordecimpunctata* exhibited a Type II functional response with a steadily declining proportion of prey consumed at higher prey density (Table 1; Figure 3). The maximum daily predation of *H. variegata* (1000 aphids) and attack rate (*a*: 1.12) was highest at 35 °C. Conversely, daily predation of *P. quatuordecimpunctata* did not differ between temperature regimes, while *a* values gradually decreased with higher temperatures (Table 1).

### 3.3. Antioxidant Responses

**Figure 4:**
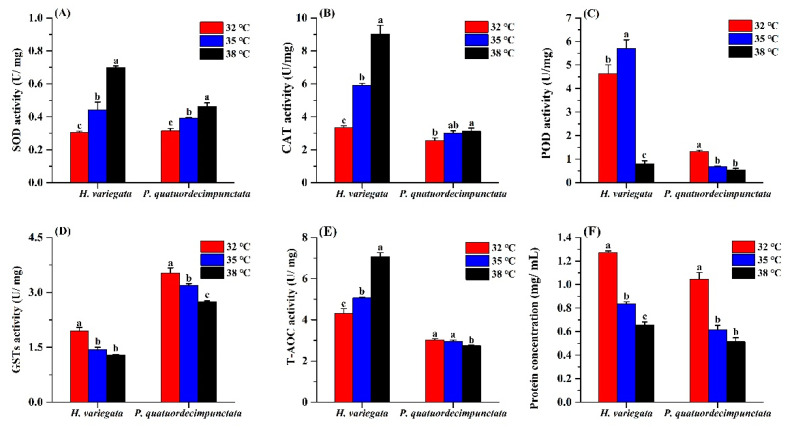
Effects of temperature stress on the antioxidant capacity of *Hippodamia variegata* and *Propylaea quatuordecimpunctata*: (**A**) SOD activity; (**B**) CAT activity; (**C**) POD activity; (**D**) GSTs activity; (**E**) T-AOC activity; (**F**) protein concentration. Different letters above the bars indicate statistically significant difference at *p* < 0.05 (ANOVA followed by a Tukey’s post hoc test).

For *H. variegata* adults held under the three temperatures for 24 h, SOD activity increased at higher temperatures (Tukey’s test: *F*_2, 6_ = 102.27, *p* < 0.001; Figure 4). Similarly, SOD activity of *P. quatuordecimpunctata* adults progressively increased with temperature (Tukey’s test: *F*_2, 6_ = 40.54, *p* < 0.001) (Figure 4A). CAT activity of *H. variegata* and *P. quatuordecimpunctata* adults also increased steadily with temperature (Tukey’s test: *F*_2, 6_ = 162.45, *p* < 0.001; *F*_2, 6_ = 6.56, *p* = 0.031, respectively) (Figure 4B). POD activity of *H. variegata* increased at 35 °C but then decreased by 82.6% at 38 °C (Tukey’s test: *F*_2, 6_ = 141.97, *p* < 0.001). Meanwhile, for *P. quatuordecimpunctata*, POD activity progressively decreased at higher temperatures (Tukey’s test: *F*_2, 6_ = 106.66, *p* < 0.001) (Figure 4C). For both ladybug species, GSTs activity consistently declined at higher temperatures (Tukey’s test: *F*_2, 6_ = 58.38, *p* < 0.001; *F*_2, 6_ = 35.91, *p* < 0.001) (Figure 4D). The T-AOC activity levels in the two ladybug adults were significantly affected by treatment temperatures. While the T-AOC activity of *H. variegata* increased with temperature (Tukey’s test: *F*_2, 6_ = 131.26, *p* < 0.001), it only declined at 38 °C for *P. quatuordecimpunctata* (Tukey’s test: *F*_2, 6_ = 9.94, *p* = 0.012) (Figure 4E). Lastly, heat stress negatively affected protein concentration in both ladybug species (Tukey’s test: *F*_2, 6_ = 457.49, *p* < 0.001; *F*_2, 6_ = 80.78, *p* < 0.001) (Figure 4F).

## 4. Discussion

Elevated temperatures affect various life history, behavioral, and physiological parameters of natural enemies, such as ladybugs [40], and thereby impact biological control to varying (and often unpredictable) extents. These impacts are species-specific and shaped by the exact range of experimental temperatures. For example, while survival of *Adalia bipunctata* (L.), *Hippodamia variegata* (Goeze), *Coccinella undecimpunctata aegyptica* (Reiche), and *Oenopia conglobata contaminata* (Menetries) is negatively affected at 32.5 °C [41], this does not apply for *Exochomus nigripennis* (Erichson), suggesting that thermotolerance of *E. nigripennis* is stronger. In this study, we demonstrated how heat stress affects adult survival, longevity, reproduction, predation rate, and the antioxidant capacity of *H. variegata* and *P. quatuordecimpunctata*. Even though both ladybug species exhibited lower survival, longevity, and fecundity under warmer conditions, this decrease—especially under a high temperature bracket—was more pronounced for *P. quatuordecimpunctata*. Hence, *P. quatuordecimpunctata* exhibited markedly lower thermal tolerance, which is in accordance with its geographical distribution patterns in northwestern China. These findings are in line with earlier work in which cold hardiness [42] and thermal tolerance are identified as key determinants of ladybug distribution [29,43]. Evidently, these functional traits determine the extent to which either ladybug species will perform under specific agro-climatic conditions [44], particularly in the harsh environments of northwestern China.

When given access to *A. gossypii* prey under varying temperatures, both predatory ladybugs exhibited a type II functional response [45,46], as also observed for other species [47,48]. Yet, even while functional response curves remain unaltered, predation rates can be subject to change under increasing temperature. For example, *Harmonia axyridis* (Pallas) predation rates of *Acyrthosiphon pisum* (Harris) nymphs increased temperature over 15–35 °C [49]. The consumption of *Harmonia dimidiata* (Fab.) to *Myzus persicae* (Sulzer) decreased with the increase in temperature (24–32 °C) [50]. In this study, the predation of *H. variegata* to *A. gossypii* was the highest at 35 °C, but the predation of *P. quatuordecimpunctata* to *A. gossypii* decreased when the temperature increased from 32 to 35 °C. Temperature-mediated differences in predation rates are highly species-dependent, with *H. variegata* consistently consuming more *A. gossypii* than *P. quatuordecimpunctata*. Yet, extrapolating these laboratory-based observations of individual predator–prey couplets to field conditions is challenging [51], as both predator and prey act within intricate, dynamic food webs in which a multitude of consumptive and non-consumptive effects (e.g., intra-specific competition and intraguild predation) dictate the ultimate outcomes [52,53,54,55]. Several of the above effects are further modulated by field-, farm-, or landscape-scale heterogeneity [56,57]. Hence, semi-field trials, manipulative experiments, and observational assays under “real-world” conditions are key to reliably predicting temperature effects on ladybug fitness or biological control.

Elevated temperatures can trigger antioxidant defenses in order to eliminate free radicals and protect the insect from thermal stress [58,59]. These antioxidant defenses are species-specific and modulated by a suite of temperature-dependent enzymes. While the activity of SOD, CAT, and GSTs in *P. japonica* increases at 39 °C, that of POD enzyme was only enhanced at 41 °C [24]. For larvae of *Mythimna separata* (Walker), the SOD, CAT, and GSTs activities were significantly raised by thermal stress (i.e., 30, 35, 40, and 45 °C) [60]. Chen et al. [61] found that the survival rate of *Ophraella communa* LeSage female adults was significantly higher than that of male adults under high temperature (i.e., 40, 42, and 44 °C) stress, and the activity of antioxidant enzymes in female adults was also higher than male adults [62]. The high survival rate of *O. communa* females may be closely related to the high antioxidative enzyme activity of females under heat stress. Our work showed variable responses of four antioxidant enzymes to increasing temperature. As SOD and CAT enzyme activity increased at higher temperatures, these compounds likely play a key role in the thermal tolerance of *H. variegata* and *P. quatuordecimpunctata*. While the activity of POD in *H. variegate* increased at 35 °C, that of POD enzyme of *P. quatuordecimpunctata* was reduced at 35 °C. Meanwhile, species-specific differences were observed in (temperature-related) T-AOC activity. In *H. variegata*, a heightened T-AOC activity at elevated temperatures could mirror a superior antioxidant capacity (and lowered likelihood of oxidative damage) than *P. quatuordecimpunctata*. These physiological patterns possibly explain the above differences in life history traits and represent the proximate causes of *H. variegata* spatiotemporal distribution.

By thus pairing (laboratory-based) life history assays with targeted recordings of antioxidant activity, our work unveils the superior thermal tolerance (and climate-adaptive capacity) of *H. variegata*. In a similar way as heat-shock proteins mediate cold hardiness and overwinter survival in coccinellids [63], the physiological adaptations to heat stress involve a suite of antioxidant enzymes. Thus, antioxidant readings can help to generate inferences regarding (coarse-grained) occurrence, seasonal abundance, and geographical distribution of individual ladybug species. However, considering how field- or landscape-level habitat modification can create more suitable micro-climatic niches, raise in-field survival rates, and thereby alter ladybug community composition [64], field-scale observational and empirical studies remain indispensable. Our experiment was performed in a constant temperature indoor environment, but the temperature of the natural was variable. Future assessments equally need to account for (climate-induced) impacts on target pests, co-occurring natural enemies, and alternating temperature. Yet, irrespective of its shortcomings, our work helps to advance (ladybug-mediated) biological control as a core constituent of climate-resilient farming systems.

## 5. Conclusions

In this study, we investigated the effects of different temperatures (32, 35, and 38 °C) on the survival, reproduction, predation, and antioxidant capacity of adult ladybugs. Our findings show how the negative impacts (on survival and reproduction) are species-specific and particularly pronounced for *P. quatuordecimpunctata*. Temperature-mediated effects are also mirrored in species’ contribution to biological control: *H. variegata’s* prey consumption rate was highest at 35 °C, while that of *P. quatuordecimpunctata* steadily decreased at higher temperatures. The above life history and biological control impacts are also reflected in the antioxidant capacity of both ladybug species, with SOD, CAT, POD, GSTs, T-AOC, and protein content all affected by temperature. Thus, laboratory assays can help to explain spatiotemporal distribution patterns of individual ladybugs and inform strategies to bolster biological control under conditions of global warming or extreme weather events.

## Figures and Tables

**Table 1:** Functional response parameters, as obtained through logistic regression, of adult female *Hippodamia variegata* and *Propylaea quatuordecimpunctata* reared on 4th instar *Aphis gossypii* under different temperature regimes. *P*_1_ is the linear coefficients of the logistic regression analysis equation; Tem is the experimental temperature; *a* is the instantaneous searching rate; *T*_h_ is the handling time. The exact structure of the functional response models is described in the text.

Species	Tem	*P*_1_ ± SE	*Na* = *aTN*_0_/(1 + *aT*_h_*N*_0_)	*R* ^2^	*a*	*T*_h_ (d)	*a*/*T*_h_	*N*_max_
*H. variegata*	32 °C	−0.0205 ± 0.0024	*Na* = 1.013*N*_0_/(1 + 0.002*N*_0_)	0.98	1.01	0.002	507	500
	35 °C	−0.0822 ± 0.0039	*Na* = 1.115*N*_0_/(1 + 0.001*N*_0_)	0.98	1.12	0.001	1115	1000
	38 °C	−0.0121 ± 0.0022	*Na* = 0.965*N*_0_/(1 + 0.002*N*_0_)	0.96	0.97	0.002	483	500
*P. quatuordecimpunctata*	32 °C	−0.0418 ± 0.0038	*Na* = 0.843*N*_0_/(1 + 0.003*N*_0_)	0.98	0.84	0.004	211	250
	35 °C	−0.0969 ± 0.0028	*Na* = 0.739*N*_0_/(1 + 0.003*N*_0_)	0.98	0.74	0.004	185	250
	38 °C	−0.0488 ± 0.0026	*Na* = 0.543*N*_0_/(1 + 0.002*N*_0_)	0.94	0.54	0.004	136	250

## Data Availability

All data analyzed in this study are included in this article.
